# Druggists and the Prescription of Poisons

**Published:** 1888-11

**Authors:** 


Druggists and the Prescription of Poisons.—The Indiana Legislature has passed a law declaring that, "From and after the passage of this Act, no pharmacist, druggist, apothecary, or other person, shall rèfill more than once prescriptions containing opium or morphine, or preparations of either, in which the dose of opium shall exceed one-fourth grain, or morphine one-twentieth grain, except with the verbal or written order of a physician." A violation of the law is declared a misdemeanor, punishable by a fine of not less than ten nor more than twenty-five dollars.

---

Cornil and Loupt have discovered a new bacterial disease affecting ducks, and designate it as *duck cholera.* The specific germ is a short bacillus, which has been cultivated, and uniformly produces a fatal disease when inocculated into ducks, but only a slight indis position when injected into other fowls.

---

The Municipal Government of Hong Kong, China, has passed a law making vaccination compulsory.

